# Ahi1 regulates serotonin production by the GR/ERβ/TPH2 pathway involving sexual differences in depressive behaviors

**DOI:** 10.1186/s12964-022-00894-4

**Published:** 2022-05-28

**Authors:** Bin Wang, Haixia Shi, Liyan Ren, Zhigang Miao, Bo Wan, Hao Yang, Xiaotang Fan, Jan-Ake Gustafsson, Miao Sun, Xingshun Xu

**Affiliations:** 1 Department of Fetology, the First Affiliated Hospital of Soochow University, Suzhou, 215004 People’s Republic of China; 2 Institute of Neuroscience, Soochow University, Suzhou, 215123 People’s Republic of China; 3 Department of Neurology, the First Affiliated Hospital of Soochow University, Suzhou, 215004 People’s Republic of China; 4 Jiangsu Key Laboratory of Neuropsychiatric Diseases, Soochow University, Suzhou, 215123 Jiangsu People’s Republic of China; 5 Department of Developmental Neuropsychology, School of Psychology, Third Military Medical University (Army Medical University), Chongqing, People’s Republic of China; 6 Department of Biosciences and Nutrition, Karolinska Institute, Huddinge, Sweden; 7 Center for Nuclear Receptors and Cell Signaling, Department of Biology and Biochemistry, University of Houston, Houston, TX USA; 8 Department of Neurology, The First Affiliated Hospital of Soochow University, Suzhou, 215006 People’s Republic of China

**Keywords:** Ahi1, Serotonin, TPH2, ERβ, GR, Estrogen

## Abstract

**Background:**

Depression is one of the most common psychiatric diseases. The monoamine transmitter theory suggests that neurotransmitters are involved in the mechanism of depression; however, the regulation on serotonin production is still unclear. We previously showed that Ahi1 knockout (KO) mice exhibited depression-like behavior accompanied by a significant decrease in brain serotonin.

**Methods:**

In the present study, western blot, gene knockdown, immunofluorescence, dual-luciferase reporter assay, and rescue assay were used to detect changes in the Ahi1/GR/ERβ/TPH2 pathway in the brains of male stressed mice and male Ahi1 KO mice to explain the pathogenesis of depression-like behaviors. In addition, E2 levels in the blood and brain of male and female mice were measured to investigate the effect on the ERβ/TPH2 pathway and to reveal the mechanisms for the phenomenon of gender differences in depression-like behaviors.

**Results:**

We found that the serotonin-producing pathway-the ERβ/TPH2 pathway was inhibited in male stressed mice and male Ahi1 KO mice. We further demonstrated that glucocorticoid receptor (GR) as a transcription factor bound to the promoter of ERβ that contains glucocorticoid response elements and inhibited the transcription of ERβ. Our recent study had indicated that Ahi1 regulates the nuclear translocation of GR upon stress, thus proposing the Ahi1/GR/ERβ/TPH2 pathway for serotonin production. Interestingly, female Ahi1 KO mice did not exhibit depressive behaviors, indicating sexual differences in depressive behaviors compared with male mice. Furthermore, we found that serum 17β-estradiol (E2) level was not changed in male and female mice; however, brain E2 level significantly decreased in male but not female Ahi1 KO mice. Further, ERβ agonist LY-500307 increased TPH2 expression and 5-HT production. Therefore, both Ahi1 and E2 regulate the ERβ/TPH2 pathway and involve sexual differences in brain serotonin production and depressive behaviors.

**Conclusions:**

In conclusion, although it is unclear how Ahi1 controls E2 secretion in the brain, our findings demonstrate that Ahi1 regulates serotonin production by the GR/ERβ/TPH2 pathway in the brain and possibly involves the regulation on sex differences in depressive behaviors.

**Video Abstract**

**Supplementary Information:**

The online version contains supplementary material available at 10.1186/s12964-022-00894-4.

## Background

Brain serotonin (5-hydroxytryptamine, 5-HT) plays an essential role in different brain functions, including modulating brain development, stress reactivity, cognition, sleep, and aggressive behaviors; the changes in brain serotonergic function may be related to different symptoms in patients with MDD that are associated with the physiological processes of serotonin [1–7]. Therefore, maintenance of brain serotonin homeostasis is critical for normal behaviors and mental health. Brain serotonin is mainly produced in the serotonergic raphe nuclei because tryptophan hydroxylase 2 (TPH2), the rate-limiting enzyme of serotonin synthesis, is highly expressed in this region. Increasing evidence indicates that TPH2 is related to MDD in depression [8–10]. A previous study demonstrated that estrogen receptor-β (ERβ) binds an estrogen response element in the promoter of TPH2 and regulates the expression of TPH2 mRNA [11], which may connect serotonin, TPH2, and ERβ. Our previous study showed that Ahi1 deficiency causes a reduction in serotonin levels in multiple brain regions and depressive behaviors in mice [12]; however, whether Ahi1 regulates brain serotonin levels through the ERβ/TPH2/serotonin pathway is unclear.

Glucocorticoid receptor (GR), a ligand-dependent regulatory transcription factor, can repress or induce the transcription of thousands of genes by directly binding to DNA response elements and/or other transcription factors [13]. In addition, our recent results indicate that AHI1 interacts directly with the C-terminus of GR to inhibit GR nuclear translocation [14]. Therefore, we speculate that GR may be involved in the regulation of serotonin by Ahi1.

Increasing evidence indicates that 17β-estradiol (E2) is involved in regulating depression, and estrogen supplementation can alleviate the depressive symptoms of patients [15, 16]. In animal studies, E2 treatment was also shown to ameliorate depressive behavior [17, 18]. In addition, there is growing evidence that E2 exerts an antidepressant effect through ERβ but not ERα [19] and that ERβ agonists also have antidepressant-like effects [20, 21]. More interestingly, the antidepressant-like effects of E2 are absent in ERβ knockout (KO) mice [22]. It has been reported that estradiol profoundly influences mood by acting on serotonergic function, but the specific mechanism is not clear [23, 24]. These data suggest that E2 may exert an antidepressant effect by regulating serotonin through ERβ.

Sex differences in vulnerability to many mental illnesses, such as depression, anxiety, alcoholism, and drug abuse, have been identified [25–28]. An increasing amount of evidence has demonstrated the existence of sex differences in major depressive disorders (MDD); there are sex differences not only in the clinical symptoms of depression [29] but also in prevalence, comorbidities, and antidepressant efficacy [30–33]. For example, the risk of depression is twice as high in adult females as in adult males [34, 35]. Interestingly, prior to puberty, there are no sex differences in the prevalence of MDD [36]. Therefore, sex differences in MDD, as the most prevalent psychiatric disorder, are becoming essential in this field, and the underlying mechanisms are being investigated to aid the development of personalized treatments for patients with MDD. Among various factors, sex differences in serotonergic function are considered a significant contributing factor to sex differences in MDD [37, 38]. Moreover, deficiencies in serotonin synthesis, transport, and degradation are modulated by sex differences [39, 40]. In addition, sex differences in gonadal hormones and HPA axis activation modulate sex differences in depression [41–44]. The process is complicated because how these factors interact is still largely unknown.

In this study, we found that Ahi1, a protein associated with susceptibility to depression [14, 45], regulated serotonin production through the GR/ERβ/TPH2 pathway, indicating a critical role for Ahi1 in the regulation of depression. Sex differences in brain estrogen levels may at least partially account for the differences in depressive behaviors between male and female Ahi1 KO mice, indicating that Ahi1 regulates serotonin production by the GR/ERβ/TPH2 pathway involving sexual differences in depression-like behaviors.

## Materials and methods

### Antibodies and reagents

ERβ (ab3576), ERα (ab32063), TPH2 (ab184505), SERT (ab102048), and Histone3 (ab1791) antibodies were purchased from Abcam Company (Abcam, MA, USA); Ahi1 (sc-515382) and GR (sc-56851) antibodies were obtained from Santa Cruz Biotechnology Inc. (Santa Cruz, CA, USA); a mouse estradiol ELISA kit was purchased from Cusabio Biological Engineering Co., Ltd. (Baltimore, MD, USA); 17β-estradiol was purchased from Sigma-Aldrich Company (St. Louis, MO, USA); a Transcriptor First Strand cDNA Synthesis Kit and 2XSYBR Green PCR Master Mix were obtained from Roche (Germany); and Lipofectamine 2000 was purchased from Invitrogen Corporation (San Diego, CA, USA). All secondary antibodies were obtained from Jackson ImmunoResearch Laboratories (West Grove, PA, USA).

### Mice

Healthy female and male C57BL/6 J mice (23–25 g) were purchased from SLAC Company (Shanghai, China). Ahi1KO mice were described previously [12, 46]. Mice were randomly housed in polypropylene cages at 22 ± 2 °C under a 12 h:12 h light–dark cycle with free access to food and water. All behavioral tests were conducted in the light phase between 9:00 and 12:00 am. The mice were brought to the testing room approximately 30 min before the test, temporarily placed in a cage after each test, and then returned to their original cage. There was an interval of at least 24 h between successive behavioral tests. All experiments were performed in accordance with the guidelines of the University Committee on Animal Care of Soochow University.

### Spatial restraint stress

A spatial restraint stress model was generated as described previously [47]. Adult C57BL/6 J mice were randomly divided into the control group and the stress group. The mice in the stress group were individually placed into a well-ventilated 50 ml centrifuge tube for 2 h (from 9:00 to 11:00) daily to restrict the movements of the mice. During spatial restraint, the mice were deprived of food and water. The mice were freely housed in their original cages in the control group but were not given access to food and water. After 2 h of spatial restraint, the mice were placed back in their original cages and given food and water. The behavioral tests were performed every 7 d.

### Forced swimming test (FST)

The FST was carried out as described previously [48]. Each mouse was placed in a 2.0 L glass beaker (diameter 10 cm, height 25 cm) filled with 14 cm of water (25 °C) and forced to swim for 6 min. Immobility time (floating while making the minimum movements necessary to keep the head above water) in 6 min was recorded. The mice were wiped with a paper towel to keep them warm and gently dried in their home cages for 30 min. After each trial, the water in the cylinder was replaced.

### Tail suspension test (TST)

The TST was performed as described previously with slight modifications [47]. In this experiment, the tail tip of each mouse was fixed to a wooden rod with adhesive tape, and the mouse was suspended 35 cm above a desk. A stopwatch was used to record the immobility time in a 6 min period. The mouse was placed back in its original cage at the end of each trial.

### Measurement of serotonin (5-HT) levels

The serotonin content in the hippocampus was measured by high-performance liquid chromatography (HPLC) as described previously [12]. Hippocampal tissues were homogenized with an ultrasonic homogenizer in 200 μl 0.4 M perchloric acid. The homogenates were centrifuged at 10,000×*g* for 15 min. After 0.4 M perchloric acid was added to the supernatants to a volume of 1 ml, the samples were then injected into the HPLC system. The serotonin levels in the samples were measured according to a serotonin standard [12, 47].

### Western blot analysis

Mice were killed, and different brain tissues, including the hippocampus and the brainstem, were collected. The samples were homogenized in lysis buffer, and the homogenates were centrifuged at 4 °C. The supernatants were collected, and the protein concentrations were determined. The protein samples (20–40 μg) were separated by 10% or 12% sodium dodecyl sulfate–polyacrylamide gel electrophoresis and subsequently transferred to nitrocellulose membranes. The membranes were blocked with 5% milk in phosphate-buffered saline/0.1% Tween 20 (PBST) for 1 h; then, the membranes were incubated with primary antibodies with shaking at 4 °C overnight. The membranes were incubated with horseradish peroxidase-conjugated secondary antibody for 1 h at room temperature on the second day. After washing, the protein signals were developed with an ECL chemiluminescence system, and densitometric analysis of the bands was carried out with Alpha Ease Image Analysis Software (version 3.1.2, Alpha Innotech).

### Immunofluorescence staining

Immunofluorescence staining was performed as previously described [49]. Mice were perfused with ice-cold saline and 4% paraformaldehyde via the heart. After the brains were immersed in 15% and 30% sucrose solution (w/v) for 24 h, the brain tissues were cut into Sects. (12 μm) with a freezing cryostat. The brain slices were attached to glass slides and washed with phosphate-buffered saline (PBS) for 30 min. The slices were incubated at room temperature with buffer containing 0.3% Triton-X-100/5% BSA/PBS for 1 h. The sections were incubated with primary antibodies overnight at 4 °C. On the second day, the sections were incubated with secondary antibodies and the nuclear dye DAPI for 1 h at 4 °C. Images were captured with a fluorescence microscope (Axio ScopeA1, Carl Zeiss, Oberkochen, Germany).

### Quantitative PCR (Q-PCR) analysis

Total RNA was isolated from brain tissues or cells by using an RNeasy Plus Mini kit according to the manufacturer’s instructions. cDNA was synthesized by reverse transcription using the Transcriptor First Strand cDNA Synthesis Kit as described previously [50, 51]. Real-time PCR was performed using a 7500 real-time PCR machine (Applied Biosystems, USA) in a volume of 20 μl, which consisted of 50 ng cDNA, 10 μl of 2XSYBR Green PCR Master Mix, and 1 μl primers (10 μM). GAPDH was used as an internal loading control. The PCR primers are listed in Additional file 1: Table S1.

### Estradiol measurement by ELISA

After the mice were killed, different brain tissues and plasma samples were collected. According to the instructions, the estradiol content in brain tissues and plasma was determined using a commercial estradiol ELISA kit (Cusabio Biological Engineering Co., Ltd., Baltimore, MD, USA).

### Plasmid construction

The ERβ promoter region was isolated by PCR amplification of human cDNA, and then the cDNAs were inserted into the pGL3-basic vector. A plasmid containing a 2-kb region of the ERβ promoter followed by a luciferase reporter gene was transfected into 293 T cells that were treated with Dex and/or GR-siRNA for 48 h. Luciferase activity was measured using a Dual-Luciferase Assay Kit (Promega, USA) according to the manufacturer's protocol. The assay results were presented as relative luciferase activity.

### Cell culture and plasmid transfection

PC12 cells or 293 T cells were maintained in DMEM (HyClone, Logan, UT, USA) containing 10% fetal bovine serum and 100 μg/ml streptomycin sulfate. When the density of the PC12 cells or 293 T cells reached approximately 50–60%, plasmid transfection was carried out. Plasmid or siRNA was mixed with 50 μl serum-free medium. Then, 2 μl Lipofectamine 2000 (Invitrogen Corporation, City, USA) was mixed with 50 μl serum-free medium. Plasmid DNA or siRNA was further mixed with Lipofectamine 2000 and incubated for 15 min at room temperature. The mixture (100 μl) was added to each well of a 6-well plate containing 900 μl serum-free medium. After 6 h, the transfection medium was removed and replaced with regular culture medium. After 48 h, cells were collected for further Western blot analysis.

### Nuclear and cytoplasmic extraction

Fresh cells or brain tissues were collected for further experiments, and the nuclear and cytoplasmic fractions were separated with a nuclear and cytoplasmic protein extraction kit (Beyotime Biotechnology, P0028, Shanghai, China). The nuclear and cytoplasmic fractions were used for further Western blot analysis.

### Administration of drugs to animals

The ERβ agonist LY500307 (Selleckchem, Houston, TX, USA) was dissolved in DMSO and freshly prepared daily before treatment. Ahi1 KO mice were randomly divided into five groups (N = 9–10 mice in each group): the vehicle-treated control group, the control treated with the ERβ agonist LY500307 (0.2 mg/kg), the vehicle-treated Ahi1 KO group, the Ahi1 KO group treated with low-dose LY500307 (0.05 mg/kg), and the Ahi1 KO group treated with high-dose LY500307 (0.2 mg/kg). The mice were treated intraperitoneally for three weeks, and behavioral tests were performed after treatment with LY500307.

### Statistical analysis

The data were presented as the mean ± SEM. Differences between two groups were analyzed by Student’s t test. Differences among groups were analyzed by one-way ANOVA followed by the LSD post hoc test or two-way ANOVA followed by Bonferroni’s multiple comparisons post hoc test where appropriate. A value of *p* < 0.05 was considered significant.

## Results

### The ERβ/TPH2/5-HT pathway was inhibited in male Ahi1 KO mice and stressed mice

**Fig. 1:**
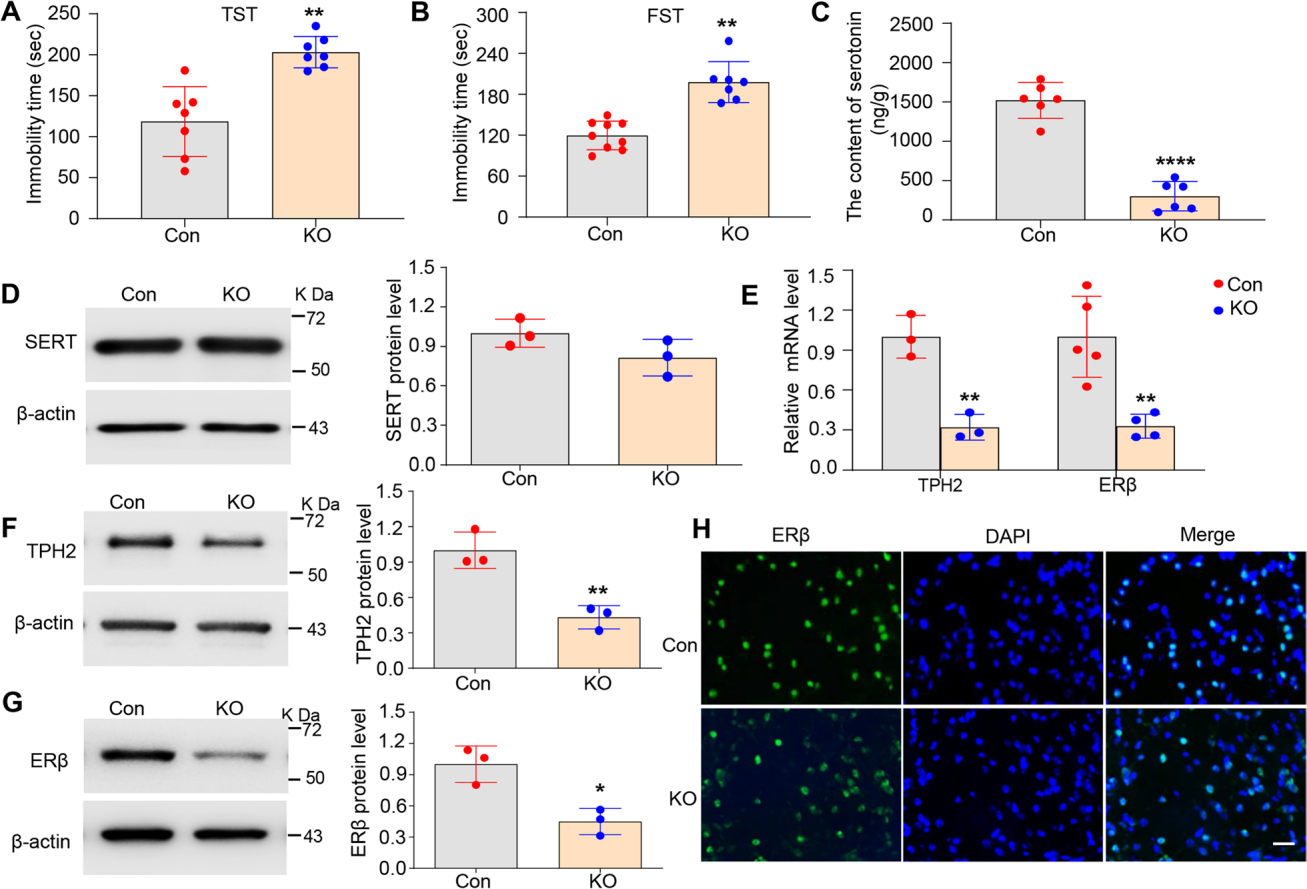
The ERβ/TPH2/5-HT pathway was down-regulated in male Ahi1 KO mice. The immobility time in TST (**A**) and FST (**B**) were performed in healthy male 2–3 month-old Ahi1 KO mice. N = 7–9. **C** The content of 5-HT was determined by HPLC in hippocampus samples from male Ahi1 KO mice. N = 6. **D** SERT protein expression was examined in hippocampus samples from male Ahi1 KO mice by Western blot analysis. N = 3. **E** TPH2 and ERβmRNA levels were quantified by quantitative PCR in the hippocampus tissues of male Ahi1 KO mice. N = 3–5. **F** TPH2 protein expression was examined in hippocampus tissues from male Ahi1 KO mice by Western blot analysis. N = 3. **G** ERβ protein expression was examined in hippocampus tissues from male Ahi1 KO mice by Western blot analysis. N = 3. **H** ERβ protein expression was detected in the brainstem in male Ahi1 KO mice by fluorescent staining. Scale bar = 20 μm. *p < 0.05; **p < 0.01; ****p < 0.0001 versus Control (Con)

Our previous studies demonstrated that Ahi1 knockout causes depressive phenotypes in male mice [12, 46, 52]. For males, the immobility time of Ahi1 KO mice was significantly increased compared with that of control mice (Fig. 1A, B). To explore the underlying mechanisms of depression-like behaviors, we measured 5-HT levels by HPLC in hippocampal tissues from male Ahi1 KO mice because previous studies have shown that serotonin is involved in the regulation of depression [12]. The 5-HT level was significantly decreased in the hippocampal tissues of male Ahi1 KO mice (Fig. 1C), suggesting that serotonin levels may cause depressive behaviors in male Ahi1 KO mice. We further explored the regulatory pathway of 5-HT production. Although MAO activity partially affects total 5-HT levels in tissues [12], determinant factors, including 5-HT transporter SERT and the limiting enzyme for 5-HT production TPH2 are related to the recycling and production of 5-HT, respectively [11]. Our findings showed that Ahi1 KO did not alter SERT expression (Fig. 1D); however, TPH2 mRNA levels in the hippocampus (Fig. 1E) and the brainstem (Additional file 2: Fig. S1B) significantly decreased in male Ahi1 KO mice. Similarly, TPH2 protein expression in the hippocampus (Fig. 1F) and the brainstem (Additional file 2: Fig. S1A) in male Ahi1 KO mice significantly decreased compared with that in control mice. Consistently, ERβ mRNA levels in the hippocampus (Fig. 1E) and brainstem (Additional file 2: Fig. S1C) were significantly reduced in male Ahi1 KO mice. ERβ protein levels in the hippocampus (Fig. 1G) and the brainstem (Additional file 2: Fig. S1D) were markedly decreased in male Ahi1 KO mice compared with male control mice. Immunofluorescence staining also confirmed the reduction in ERβ protein expression in the male Ahi1 KO mouse brain (Fig. 1H), but the ERβ positive cells had no difference between the two groups (Additional file 2: Fig. S1F). The protein expression of ERα, an important isoform of ER, also did not change in the hippocampi of Ahi1 KO mice (Additional file 2: Fig. S1E). Therefore, the ERβ/TPH2 pathway was affected in male Ahi1 KO mice.

To further confirm that the ERβ/TPH2/5-HT pathway is also changed in male stressed mice, spatial restraint stress was used to induce depressive behaviors in mice. The immobility time in TST and FST was significantly increased for male stressed mice after spatial restraint stress for 2 w compared with control male mice (Additional file 3: Fig. S2A, B). Similarly, TPH2 mRNA and protein levels (Additional file 4: Fig. S3A, B) were also decreased markedly in the hippocampus in male stressed mice compared with male control mice. ERβ mRNA and protein (Additional file 4: Fig. S3C, D) levels were significantly reduced in male stressed mice. SERT expression in male stressed mice also did not change (Additional file 4: Fig. S3E). Importantly, Ahi1 protein expression decreased significantly in male stressed mice (Additional file 3: Fig. S2C), suggesting that Ahi1 is critical for the ERβ/TPH2/5-HT pathway in regulating depressive behaviors.

### Ahi1 positively regulated ERβ expression

**Fig. 2:**
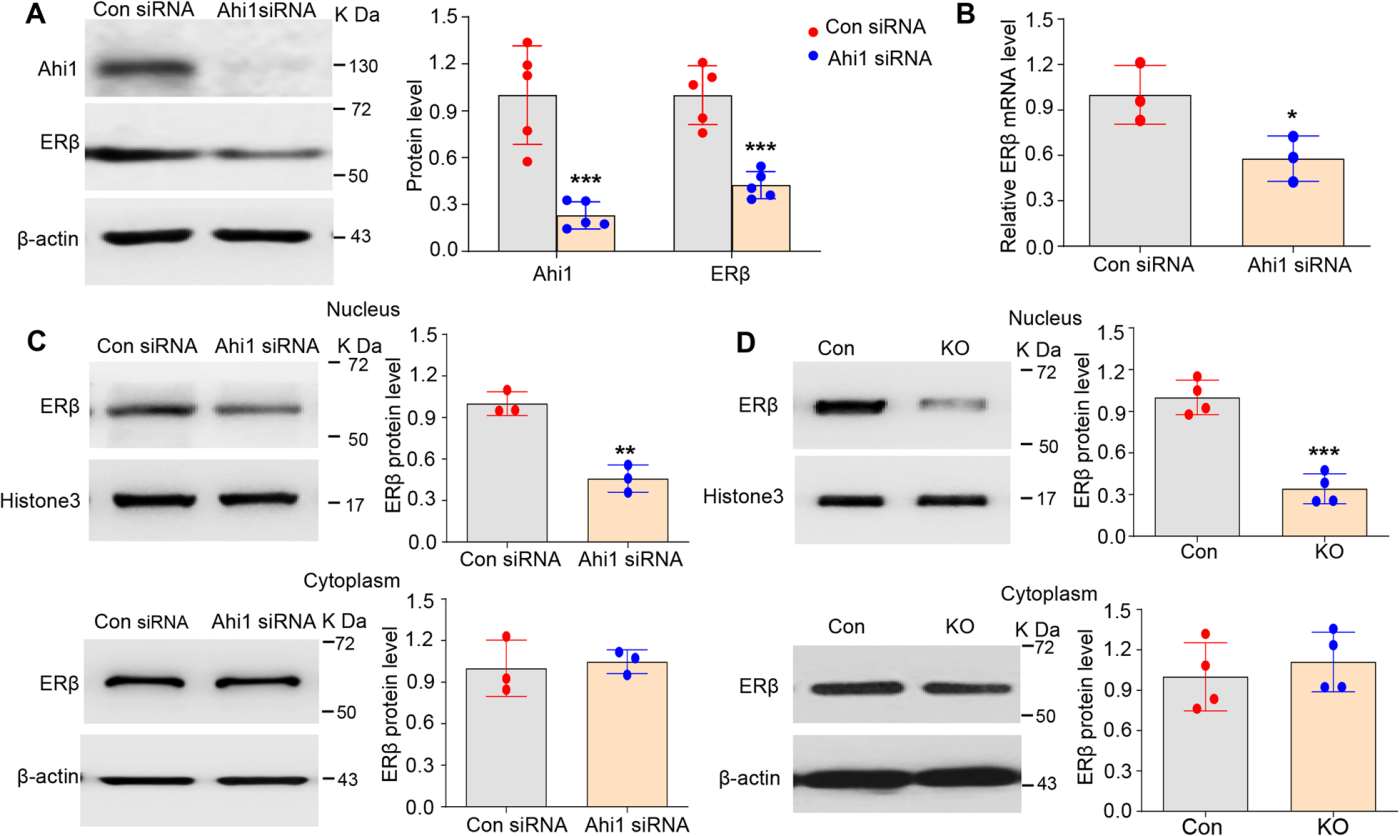
Ahi1 positively regulated ERβ expression. **A,** Ahi1 and ERβ protein expression were examined in Ahi1-siRNA transfected PC12 cells 48 h by Western blot analysis. N = 5. **B** ERβ mRNA level in Ahi1-siRNA transfected PC12 cells 48 h was examined by quantitative PCR Quantitative analysis. N = 3. **C** ERβ protein expression was detected in the cytoplasm and nucleus of Ahi1-siRNA transfected PC12 cells 48 h by Western blot analysis. N = 3. **D** ERβ protein expression was detected in cytoplasm and nucleus in the hippocampus tissue from male Ahi1 KO mice by Western blot analysis. N = 4. *p < 0.05; **p < 0.01; ***p < 0.001 versus Control

To examine the regulatory effect of Ahi1 on the ERβ/TPH2/5-HT pathway, we knocked down Ahi1 in PC12 cells and examined ERβ expression. After Ahi1-siRNA transfection for 48 h, Ahi1 was significantly knocked down (Fig. 2A). ERβ protein (Fig. 2A) and mRNA levels (Fig. 2B) were both significantly decreased. Because ERβ is one of the classical nuclear receptors of estrogen and is mainly distributed in the nucleus [53, 54], we further determined the effect of Ahi1 on cellular ERβ distribution. Ahi1 knockdown reduced ERβ expression in the nucleus but not in the cytoplasm (Fig. 2C). Consistently, male Ahi1 KO mice showed a significant decrease in nuclear ERβ expression in the hippocampus; however, cytoplasmic ERβ expression was not significantly changed by Ahi1 KO (Fig. 2D). These findings suggested that Ahi1 knockdown or KO not only decreased ERβ expression but also reduced the nuclear translocation of ERβ, hindering its functions as a nuclear receptor.

### GR inhibited the expression of ERβ by binding to the promoter of ERβ

**Fig. 3:**
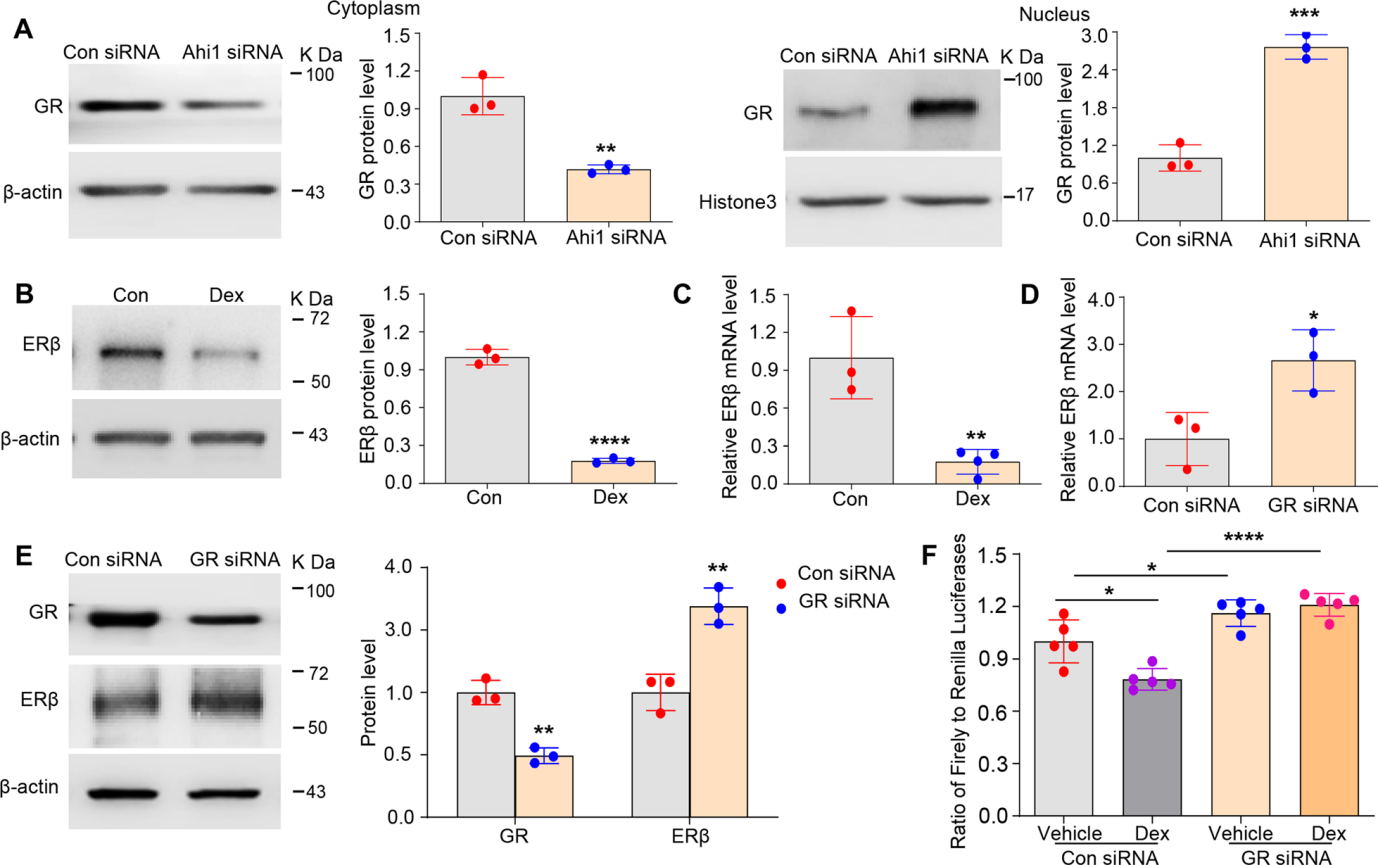
GR inhibited the expression of ERβ by binding to the promoter of ERβ. **A** GR protein expression was detected in the cytoplasm and nucleus of Ahi1-siRNA transfected PC12 cells by Western blot analysis. N = 3. **B** ERβ protein expression was detected in Dex treated PC12 cells by Western blot analysis. N = 3. **C** ERβ mRNA levels in Dex treated PC12 cells were examined by quantitative PCR Quantitative analysis. N = 3–4. **D** ERβ mRNA levels in GR-siRNA transfected PC12 cells were examined by quantitative PCR Quantitative analysis. N = 3. **E** GR and ERβ protein expression were examined in GR-siRNA transfected PC12 cells by Western blot analysis. N = 3. **F** PGL3-basic-ERβ-promoter Plasmid transfected 293 T cells were treated with Dex and/or GR-siRNA, and the luciferase activity was measured using a Dual-Luciferase Assay kit. N = 5. *p < 0.05; **p < 0.01; ****p < 0.0001 versus Control

Previous studies have demonstrated that Ahi1 is expressed predominantly in the cytoplasm, neuronal processes, and sigmoid bodies but is found at lower levels in the nucleus [55, 56]. Therefore, we speculated that Ahi1 might regulate ERβ transcription indirectly by interacting with some transcription factors. Our recent results indicated that Ahi1 interacts directly with glucocorticoid receptor (GR), an effector of the HPA system, and inhibits GR nuclear translocation [14]. However, it is still unclear whether Ahi1 regulates ERβ expression through the transcriptional effects of GR. Therefore, we examined the cellular distribution of GR after Ahi1-siRNA transfection. GR protein expression was significantly reduced in the cytoplasm and increased in the nucleus (Fig. 3A), suggesting that Ahi1 has an inhibitory effect on GR nuclear translocation. We further examined the regulatory effect of increased GR nuclear translocation on ERβ expression. PC12 cells were treated with dexamethasone (Dex, an agonist of GR that promotes GR nuclear translocation). After Dex treatment for 72 h, ERβ protein (Fig. 3B) and mRNA (Fig. 3C) levels decreased significantly. Interestingly, nuclear ERβ protein expression was significantly reduced (Additional file 5: Fig. S4A), but cytoplasmic ERβ protein expression did not change (Additional file 5: Fig. S4B). In contrast, after GR was knocked down by GR-siRNA in PC12 cells, ERβ mRNA (Fig. 3D) and protein (Fig. 3E) levels increased significantly. Therefore, the results showed that increased GR nuclear translocation inhibited the transcription of ERβ mRNA.

To clearly demonstrate the direct regulatory effect of GR on ERβ mRNA transcription, we constructed a plasmid that contained a 2-kb region of the promoter of ERβ followed by a luciferase reporter gene. Plasmid-transfected 293 T cells were treated with Dex and/or GR-siRNA. In control-siRNA treated cells, luciferase expression was significantly reduced in cells treated with Dex compared with vehicle-treated cells (lane 1 and lane 2; Fig. 3F); however, compared with vehicle/control-siRNA, GR-siRNA increased luciferase expression (lane 1 and lane 3; Fig. 3F). Interestingly, Dex treatment did not reverse the GR knockdown-induced increase in luciferase expression (lane 3 and lane 4; Fig. 3F). A comparison of the two Dex-treated groups revealed that GR knockdown increased reporter gene expression (lane 2 and lane 4; Fig. 3F). These data demonstrated that GR could interact with the ERβ promoter and inhibits the transcription of ERβ mRNA.

### An ERβ agonist reversed depression-like behaviors and increased TPH2 and serotonin production in male Ahi1 KO mice

**Fig. 4:**
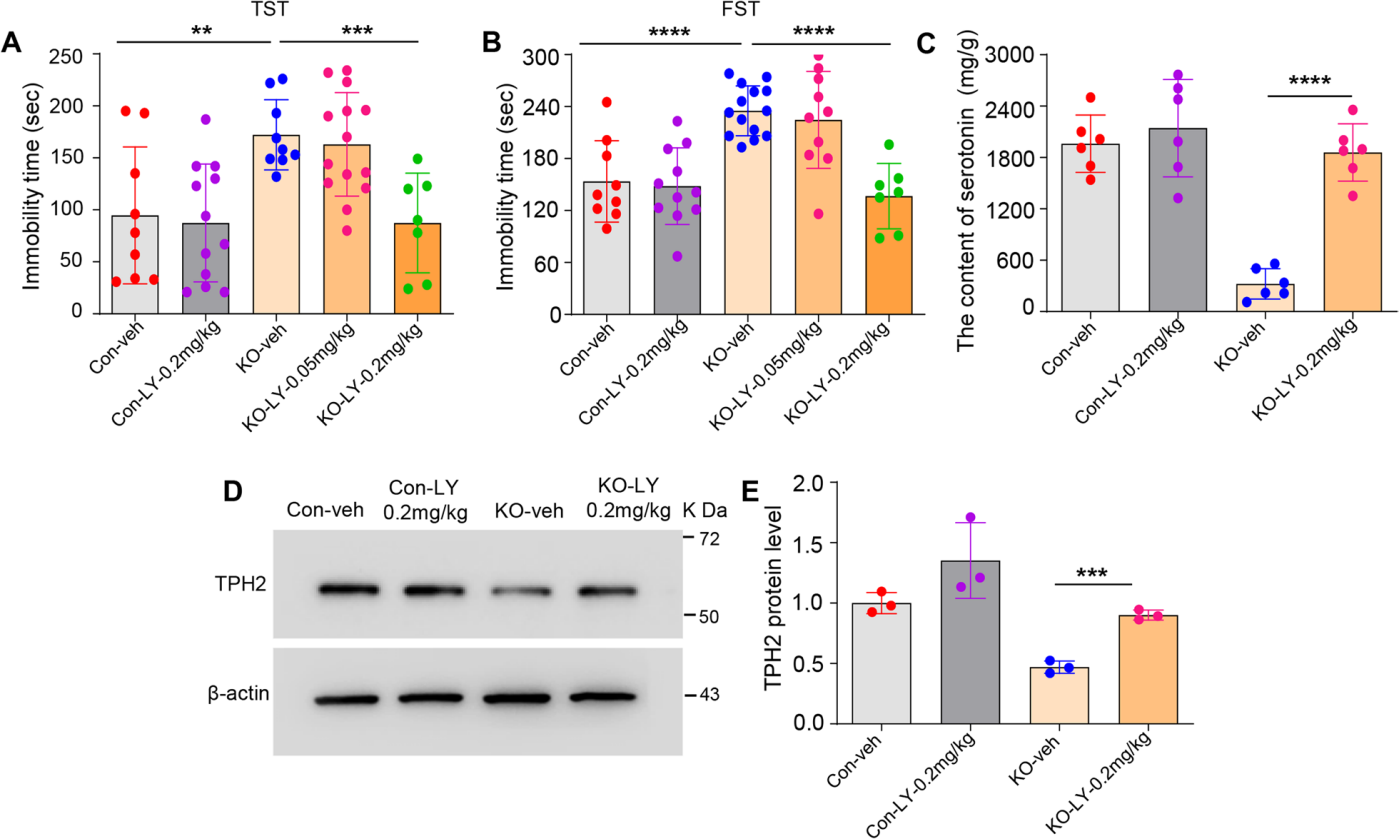
ERβ agonist (LY-500307) reversed depression-like behaviors and increased TPH2 and serotonin production in male Ahi1 KO mice. **A**, **B** After 3 weeks treatment with exogenous ERβ agonist, behavioral tests were performed, the immobility time in TST and FST was recorded. N = 7–14. **C** After 3 weeks treatment with an exogenous ERβ agonist, the content of 5-HT in the hippocampus samples from male mice was determined by HPLC. N = 6. **D**, **E** TPH2 protein expression in the hippocampus samples from male Ahi1 KO mice was examined by Western blot analysis. N = 3. **p < 0.01; ***p < 0.001; ****p < 0.0001

Previous studies have demonstrated that ERβ agonists ameliorate depressive behaviors [20, 21, 57] and that the antidepressant-like effects of E2 are absent in ERβ knockout mice [22]. It is unclear whether ERβ agonists rescue depression-like behaviors in Ahi1 KO mice. Consistent with our previous study, male Ahi1 KO mice showed longer immobility times than control mice in the TST and FST (Fig. 4A, B). After treatment with the ERβ agonist LY-500307 (LY, 0.05 mg-0.2/kg/day) for 3 weeks, LY treatment at a higher dosage (0.2 mg/kg/day) had a significant antidepressant effect in the TST and FST (Fig. 4A, B). Compared with vehicle treatment, LY (0.2/kg/day) treatment reversed the decrease in 5-HT content (Fig. 4C) and TPH2 protein expression (Fig. 4D, E) in male Ahi1 KO mice.

### The ERβ/TPH2/5-HT pathway was not changed in female Ahi1 KO mice and stressed mice

**Fig. 5:**
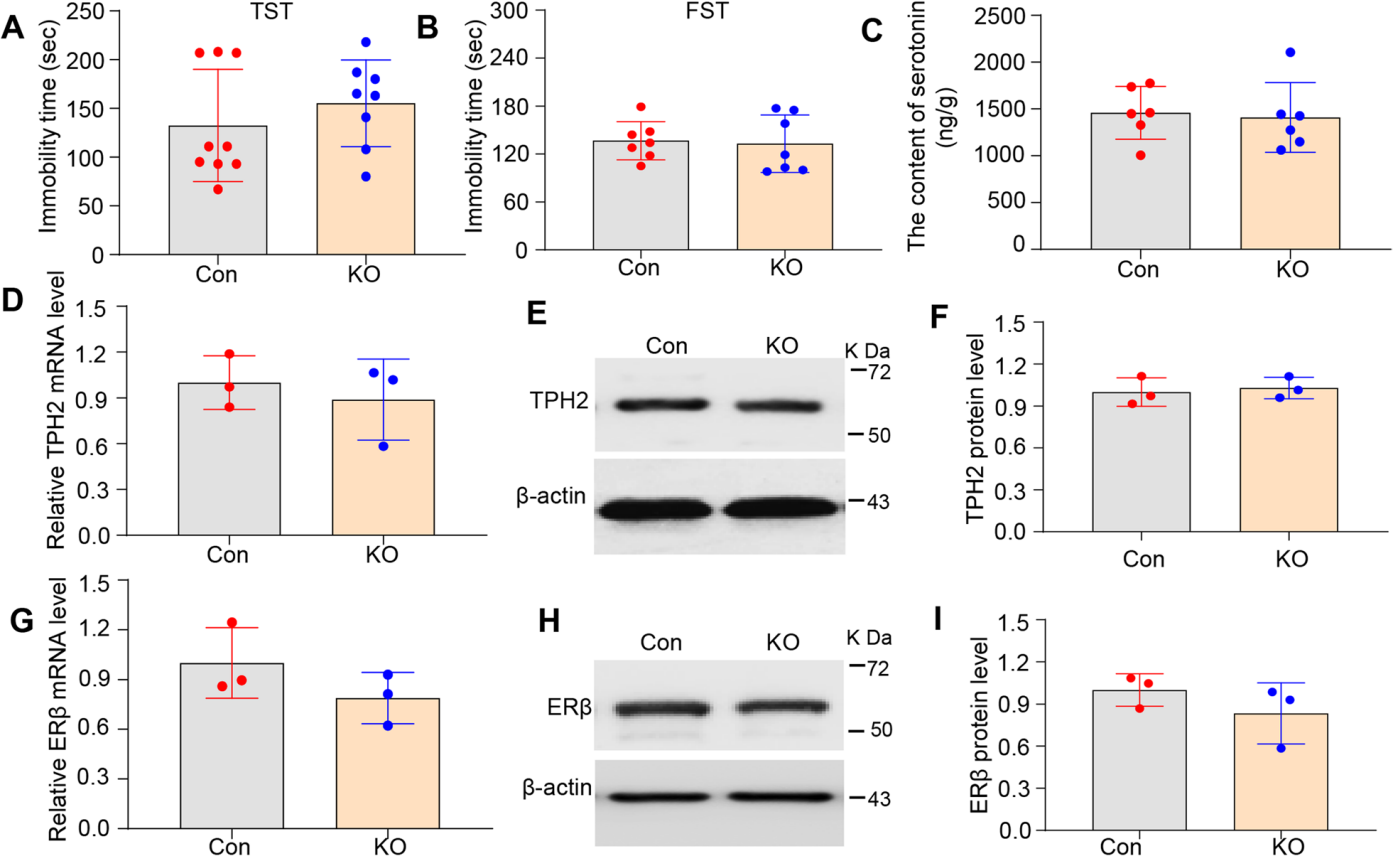
The ERβ/TPH2/5-HT pathway was not changed in female Ahi1 KO mice. The immobility time in TST (**A**) and FST (**B**) were performed in healthy female 2–3 month-old Ahi1 KO mice. N = 7–9. **C** The content of 5-HT was determined by HPLC in hippocampus samples from female Ahi1 KO mice. N = 6. **D** TPH2 mRNA level was quantified by quantitative PCR in the hippocampus tissues from female Ahi1 KO mice. N = 3. **E**, **F** TPH2 protein expression was examined in hippocampus tissues from female Ahi1 KO mice by Western blot analysis. N = 3. **G** ERβ mRNA level was quantified by quantitative PCR in the hippocampus tissues from female Ahi1 KO mice. N = 3. **H**, **I** ERβ protein expression was examined in hippocampus tissues from female Ahi1 KO mice by Western blot analysis. N = 3

To show the effect of Ahi1 KO on depressive phenotypes in mice of different sexes, we also subjected female Ahi1 KO mice to behavioral tests, including the TST and FST. Interestingly, for females, the immobility time of Ahi1 KO mice was not different from that of control mice (Fig. 5A, B), indicating that there were sex differences in depressive phenotypes in Ahi1 KO mice. To explore the underlying mechanisms of the sex differences, we measured 5-HT levels by HPLC in hippocampal tissues from female Ahi1 KO mice because previous studies have shown that serotonin is involved in regulating sex differences in depression [58], hippocampal 5-HT levels in female Ahi1 KO mice were not different from those in control mice (Fig. 5C), consistent with its normal phenotype. This suggested that the difference in serotonin levels between the sexes may cause sex differences in depressive behaviors in Ahi1 KO mice. We further explored the changes in ERβ/TPH2/5-HT pathway in female Ahi1 KO mice. Our results showed no changes in TPH2 mRNA level and protein expression in the hippocampus (Fig. 5D–F) and the brainstem (Additional file 2: Fig. S1A, B) in female AHI1 KO mice. Consistently, ERβ mRNA level and protein expression in the hippocampus (Fig. 5G–I) and brainstem (Additional file 2: Fig. S1C, D) were also unaltered in female Ahi1 KO mice. Therefore, the ERβ/TPH2/5-HT pathway was affected in female Ahi1 KO mice.

To further confirm whether the ERβ/TPH2/5-HT pathway is also unchanged in female stressed mice, spatial restraint stress was used to induce depressive behaviors in mice. The immobility time of female stressed mice was not different from that of female control mice (Additional file 3: Fig. S2A, B). In order to explain this sex difference, we measured Ahi1 protein and ERβ/TPH2/5-HT pathway. The Ahi1 protein level (Additional file 3: Fig. S2D) did not change in female stressed mice compared with male control mice. Similarly, TPH2 mRNA and protein levels (Additional file 4: Fig. S3A, B) were not altered in the hippocampus in female stressed mice compared with male control mice; ERβ mRNA and protein (Additional file 4: Fig. S3C, D) levels were also unchanged in female stressed mice. These results showed that the normal phenotype and 5-HT content of female AHI1 KO mice and stressed mice were not only affected by Ahi1/GR/ERβ/TPH2/5-HT pathway regulation but may also be related to other pathways.

### Brain E2 expression was reduced in male Ahi1 KO mice but not in female Ahi1 KO mice.

**Fig. 6:**
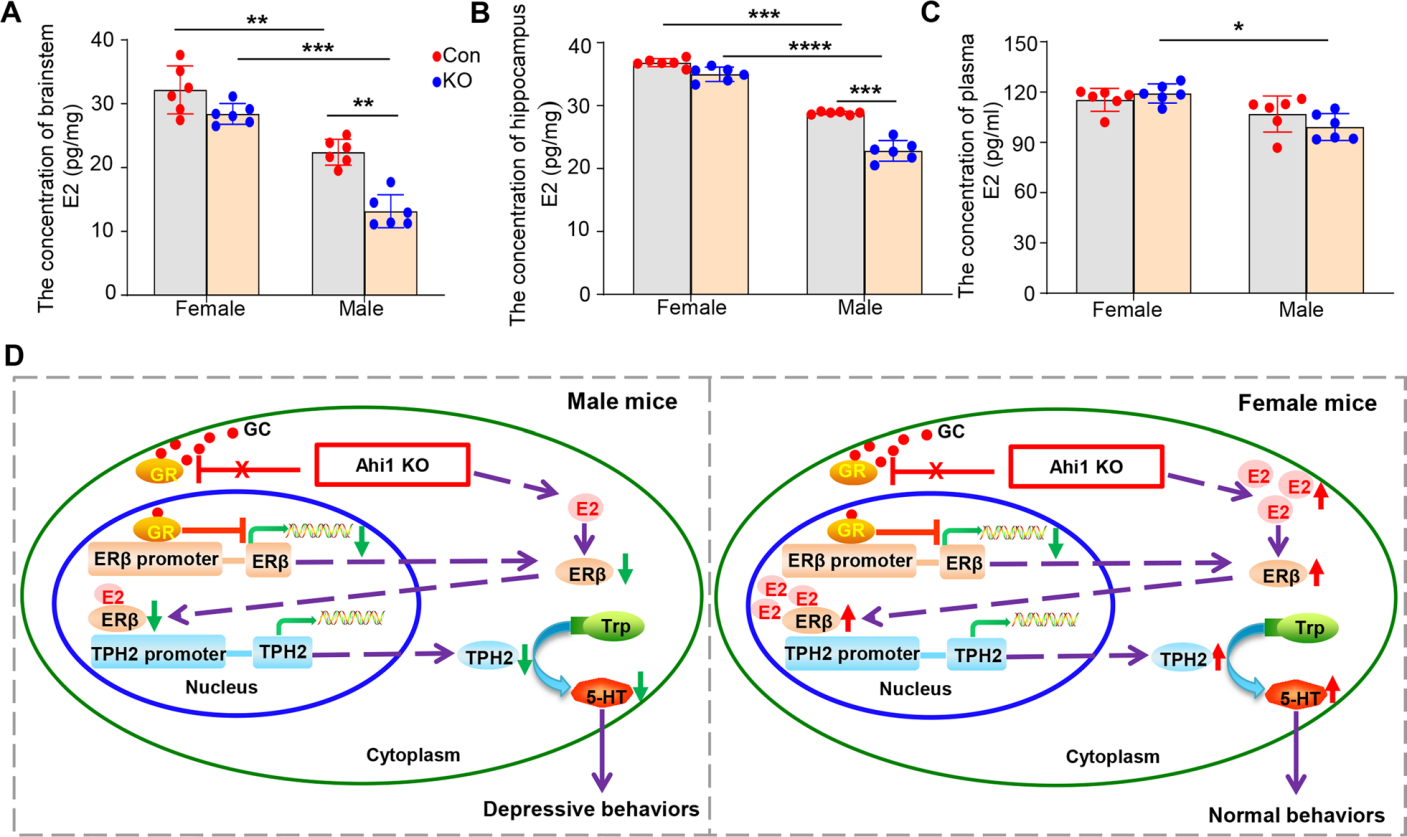
Brain E2 was reduced in male Ahi1 KO mice but not in female Ahi1 KO mice. **A**, **B** The content of E2 in the brainstem (**A**) and the hippocampus (**B**) from male and female Ahi1 KO mice was detected by ELISA kit. N = 6. **C** The content of E2 in plasma from female Ahi1 KO mice and male Ahi1 KO mice was also detected by an ELISA kit. N = 6. **D** The proposed diagram of the Ahi1 regulates serotonin production by the GR/ERβ/TPH2 pathway involved in sex differences in depressive behavior. In male Ahi1 KO mice, a reduction in Ahi1 expression decreases the cytoplasmic GR level, promotes ligand-dependent GR nuclear translocation and ERβ/TPH2/5-HT pathway was down-regulated; the lower E2 content in male mice could not compensate for the decrease in ERβ expression and depressive behavior in male Ahi1 KO mice; In female Ahi1 KO mice, high levels of E2 promoted ERβ protein expression and compensated for the Ahi1 deficiency-induced reduction in ERβ protein expression. Finally, these changes caused an increase in TPH2 and 5-HT levels, leading to normal behaviors in female Ahi1 KO mice. *p < 0.05; **p < 0.01; ***p < 0.001; ****p < 0.0001

Due to the therapeutic effect of the ERβ agonist LY in male Ahi1 KO mice, we explored whether the estrogen level in the brain or plasma affects sex differences in depressive behaviors. The contents of E2 in the brainstem and the hippocampus were not different between female Ahi1 KO mice and female control mice (Fig. 6A, B); however, the content of E2 in the brainstem and the hippocampus was significantly decreased in male Ahi1 KO mice compared with male control mice (Fig. 6A, B). The content of brain E2 was significantly higher in female mice than in male control mice (Fig. 6A, B). Interestingly, E2 levels in plasma were not different between female and male Ahi1 KO mice and their corresponding controls (Fig. 6C), and E2 levels in plasma were only slightly higher in female Ahi1 KO mice than in male Ahi1 KO mice (Fig. 6C). Therefore, our findings suggested that endogenous E2 in the brain may also be involved in the regulation of depressive behaviors through the ERβ/TPH2/5-HT pathway. In male Ahi1 KO mice, the reduction in 5-HT levels can be easily explained by the Ahi1/GR/ERβ/TPH2/5-HT pathway; however, this pathway does not explain the expected levels of ERβ, TPH2, and 5-HT in the brain tissues of female Ahi1 KO mice. Sex differences were explained by increased E2 levels in the brains of female Ahi1 KO mice because E2 may compete with GR for the ERβ promoter and alleviate the inhibition of the ERβ transcription by GR.

## Discussion

Our findings demonstrate that Ahi1 regulates ERβ protein expression by affecting GR nuclear translocation. Conversely, a reduction in Ahi1 expression decreases the cytoplasmic GR level, promotes ligand-dependent GR nuclear translocation and decreases the protein expression of ERβ in the nucleus; however, E2 can increase ERβ protein levels. Therefore, in female Ahi1 KO mice, high levels of E2 promoted ERβ protein expression and compensated for the Ahi1 deficiency-induced reduction in ERβ protein expression. Finally, these changes caused an increase in TPH2 and 5-HT levels, leading to antidepressant behavior. However, the lower E2 content in male mice could not compensate for the decrease in ERβ expression caused by Ahi1 KO, which led to a decrease in TPH2 and 5-HT levels and depressive behavior in male Ahi1 KO mice (Fig. 6D).

5-HT is a neurotransmitter that appears early in development and is widely distributed throughout the brain, and plays a central role in brain development, mood regulation, stress response, and risk of psychiatric disorders [1, 4, 7, 59]. Our previous findings suggested that 5-HT in the brain contributes to the depression-like behaviors induced by Ahi1 deficiency [12]. Our recent results indicated that Ahi1 inhibits GR nuclear translocation by directly interacting with GR [14]. More interestingly, the expression of ERβ decreased in the brains of male Ahi1 KO mice, and the reduction in expression was mainly in the nucleus, not the cytoplasm. Both GR and ER are ligand-responsive transcription factors that belong to the nuclear receptor superfamily [60]. Therefore, we speculated that Ahi1 might regulate ERβ transcription by interacting with GR. In Dex and/or GR-siRNA treated PC12 cells, luciferase reporter assays proved that Ahi1 inhibits GR nuclear translocation and regulates ERβ transcription; however, whether GR affects ERβ activity needs further investigation. The protein levels of 5-HT transporter SERT and the ERα were not changed in male Ahi1 KO and the mouse model of chronic restraint stress-induced depression-like behaviors; therefore, they may not be responsible for the decline in 5-HT levels and depression-like behavior in depressed mice. The ERβ/TPH2/5-HT pathway was inhibited in male Ahi1 KO mice and stressed mice, suggesting that the pathway has a critical role in regulating depressive behaviors. However, there was no change in the ERβ/TPH2/5-HT pathway or depression-like behavior in female Ahi1 KO and chronic restraint stressed mice, implying that there may be other signaling pathways involved in the regulation of 5-HT and behavior in female Ahi1 KO and chronic restraint stress mice.

Accumulating evidence suggests that there are sex differences in depression symptoms and the response to antidepressant treatment [33, 61]. It is clear that the prevalence of depression is higher in women than in men. In animals, male rats and mice seem to be more susceptible than females to depression-like effects [62, 63]. Sex differences are related to many factors, including artifacts and social or biological factors [64]. Our study used male and female Ahi1 KO and a mouse model of chronic restraint stress-induced depression-like behaviors to investigate the mechanisms underlying the decrease in 5-HT content-induced depression-like behaviors and sex differences, explaining the causes of sex differences from a biological perspective.

Estrogen has been used successfully to treat depression or depression-like behaviors in humans and rodents [15, 18, 65, 66]. Interestingly, E2-treated spayed macaques exhibited increased TPH mRNA expression in the dorsal raphe nucleus, indicating that E2 induces TPH gene expression in nonhuman primates [67]. In our study, the content of endogenous brain E2 was significantly higher in female mice than in male control mice, and the brain content of E2 was significantly decreased in male Ahi1 KO mice compared with male control mice; however, there was no difference between female Ahi1 KO mice and female control mice (Fig. 6A, B). Interestingly, E2 levels in plasma were not different between male Ahi1 KO mice and control mice (Fig. 6C). Therefore, brain E2 may play an antidepressant role by influencing TPH gene expression, leading to sex differences in depressive-like mouse models. These results suggested that E2 is involved in the regulation of depressive behaviors through the ERβ/TPH2/5-HT pathway and that the E2 level in the brain may be an important biochemical marker for the diagnosis of depression.

## Conclusions

Ahi1 modulates the ERβ/TPH2/5-HT pathway through modulating GR nuclear translocation. The ERβ/TPH2/5-HT pathway is inhibited in male Ahi1 KO and chronic restraint stress-induced depressed mice but not in female mice. The difference in endogenous brain estrogen levels causes sex differences in depressive behaviors. Therefore, Ahi1 regulates serotonin production by the GR/ERβ/TPH2 pathway involving sex differences in depression-like behaviors; our findings may facilitate finding personalized treatments for patients with MDD.

## Supplementary Information

**Additional file 1: Table S1.** Primers used in this study.

**Additional file 2: Fig. S1. **There were sex differences in ERβ/TPH2/5-HT pathway and depression-like behaviors in the brainstem of Ahi1 KO mice. **A** After female and male Ahi1 KO mice were sacrificed, brainstem tissues were collected, and TPH2 protein expression was examined by Western blot analysis. N=3. **B** TPH2 mRNA levels were quantified by quantitative PCR in the brainstem tissues of female and male Ahi1 KO mice. N=3. **C **ERβ mRNA expression in brainstem tissue of female and male Ahi1 KO mice was examined by quantitative PCR. N=3-5. **D **ERβ protein expression in brainstem tissue of female and male Ahi1 KO mice was examined by Western blot analysis. N=3. **E **ERα protein expression was examined in hippocampus tissue of male Ahi1 KO mice by Western blot analysis. N=3. **F** Quantity analysis of ERβ expression fluorescence intensity and the number of ERβ-positive cells in the brainstem in male Ahi1 KO mice. *p<0.05, **p<0.01, ***p<0.001 versus Control.

**Additional file 3: Fig. S2. **Stress led to depression-like behaviors and a decrease of Ahi1 in the hippocampus of male mice, but not in female mice. **A**, **B** After the healthy female and male C57 mice were stressed for 2 weeks, the behavioral tests were performed in the stressed mice. the immobility time in TST and FST tests was recorded. N=8-11. **C, D** After female and male C57 mice were stressed for 2 weeks, the hippocampal tissues of male and female stressed mice were collected and their Ahi1 content was detected by Western blot. N=4. **p<0.01, ***p<0.001 versus Control).

**Additional file 4: Fig. S3.** There were sex differences in ERβ/TPH2/5-HT pathway in stressed mice. **A**, **B** After female and male stressed mice were sacrificed, hippocampus tissue was collected, TPH2 mRNA levels were quantified by quantitative PCR (**A**) and TPH2 protein expression was examined by Western blot analysis (**B**). N=3. **C** ERβ mRNA levels were quantified by quantitative PCR in the hippocampus tissue from female and male stressed mice. N=3-4. **D** ERβ protein expression was performed in hippocampus tissue from female and male stressed mice. N=3. **E** SERT protein expression was examined in the hippocampus tissue of male stressed mice by Western blot analysis. N=3. **p<0.01 versus Control.

**Additional file 5: Fig. S4. **Dexamethasone promotes GR nuclear translocation and inhibited the transcription of ERβ mRNA. ERβ and GR protein expression were detected in the nucleus (A) and cytoplasm (B) in Dex-treated PC12 cells for 72 h by Western blot analysis. N=3. **p<0.01, ***p<0.001 versus Control.

## Data Availability

All data generated or analyzed during this study are included in this published article.

## Abbreviations

5-HT: 5-Hydroxytryptamine
E2: 17β-Estradiol
ERβ: Estrogen receptor-β
FST: Forced swimming test
GR: Glucocorticoid receptor
HPLC: High-performance liquid chromatography
KO: Knockout
MDD: Major depressive disorders
PBS: Phosphate-buffered saline
TPH2: Tryptophan hydroxylase 2
TST: Tail suspension test
