# Supplementary material for: Ahi1 regulates serotonin production by the GR/ERβ/TPH2 pathway involving sexual differences in depressive behaviors
Source: Cell Commun Signal. 2022 May 28;20:74. doi: 10.1186/s12964-022-00894-4 (PMC9148486; doi:10.1186/s12964-022-00894-4)
Supplement: Supplementary file 2 — Additional file 1: Table S1. Primers used in this study. [file 12964_2022_894_MOESM2_ESM.docx]

**Ahi1 regulates serotonin production by the GR/ERβ/TPH2 pathway involving** **sexual differences in depressive behaviors**

Bin Wang^1,2^, Haixia Shi^2^, Liyan Ren^2^, Zhigang Miao^2^, Bo Wan^2^, Hao Yang^1^, Xiaotang Fan^5^, Jan-Ake Gustafsson^6,7^, Miao Sun^1^, Xingshun Xu^2,3,4^

^1^Department of Fetology, the First Affiliated Hospital of Soochow University, Suzhou, 215004, China; ^2^Institute of Neuroscience, Soochow University, Suzhou, 215123, China; ^3^Department of Neurology, the First Affiliated Hospital of Soochow University, Suzhou, 215004, China; ^4^Jiangsu Key Laboratory of Neuropsychiatric Diseases, Soochow University, Suzhou, Jiangsu 215123, China; ^5^Department of Developmental Neuropsychology, School of Psychology, Third Military Medical University (Army Medical University), Chongqing, PR China; ^6^Department of Biosciences and Nutrition, Karolinska Institute, Huddinge, Sweden; ^7^Center for Nuclear Receptors and Cell Signaling, Department of Biology and Biochemistry, University of Houston, Houston, TX, USA.

Bin Wang and Haixia Shi contributed equally.

***Correspondence authors:**

**Xingshun Xu MD, PhD**

Department of Neurology, the First Affiliated Hospital of Soochow University, Suzhou, 215006, China

Email: xingshunxu@suda.edu.cn

Telephone: 86-512-65883252

**Running title:** Ahi1 regulates depressive behaviors

**Fig S1** There were sex differences in ERβ/TPH2/5-HT pathway and depression-like behaviors in the brainstem of Ahi1 KO mice. **A** After female and male Ahi1 KO mice were sacrificed, brainstem tissues were collected, and TPH2 protein expression was examined by Western blot analysis. N=3. **B** TPH2 mRNA levels were quantified by quantitative PCR in the brainstem tissues of female and male Ahi1 KO mice. N=3. **C** ERβ mRNA expression in brainstem tissue of female and male Ahi1 KO mice was examined by quantitative PCR. N=3-5. **D** ERβ protein expression in brainstem tissue of female and male Ahi1 KO mice was examined by Western blot analysis. N=3. **E** ERα protein expression was examined in hippocampus tissue of male Ahi1 KO mice by Western blot analysis. N=3. **F** Quantity analysis of ERβ expression fluorescence intensity and the number of ERβ-positive cells in the brainstem in male Ahi1 KO mice. *p<0.05, **p<0.01, ***p<0.001 versus Control.

**Fig S2** Stress led to depression-like behaviors and a decrease of Ahi1 in the hippocampus of male mice, but not in female mice. **A**, **B** After the healthy female and male C57 mice were stressed for 2 weeks, the behavioral tests were performed in the stressed mice. the immobility time in TST and FST tests was recorded. N=8-11. **C, D** After female and male C57 mice were stressed for 2 weeks, the hippocampal tissues of male and female stressed mice were collected and their Ahi1 content was detected by Western blot. N=4. **p<0.01, ***p<0.001 versus Control.

**Fig S3** There were sex differences in ERβ/TPH2/5-HT pathway in stressed mice. **A**, **B** After female and male stressed mice were sacrificed, hippocampus tissue was collected, TPH2 mRNA levels were quantified by quantitative PCR (**A**) and TPH2 protein expression was examined by Western blot analysis (**B**). N=3. **C** ERβ mRNA levels were quantified by quantitative PCR in the hippocampus tissue from female and male stressed mice. N=3-4. **D** ERβ protein expression was performed in hippocampus tissue from female and male stressed mice. N=3. **E** SERT protein expression was examined in the hippocampus tissue of male stressed mice by Western blot analysis. N=3. **p<0.01 versus Control.

**Fig S4 Dexamethasone promotes GR nuclear translocation and inhibited the transcription of ERβ mRNA**. ERβ and GR protein expression were detected in the nucleus (**A**) and cytoplasm **(B)** in Dex-treated PC12 cells for 72 h by Western blot analysis. N=3. **p<0.01, ***p<0.001 versus Control.

**Table S1**

**Primers used in this study.**

| **Gene name** | **Forward** | **Reverse** |
| --- | --- | --- |
| ERβ (mice) | CTGTGCCTCTTCTCACAAGGA | TGCTCCAAGGGTAGGATGGAC |
| TPH2(mice) | CCCAAGTTCGCTCAGTTTTC | CACACGCCTTGTCAGAAAGA |
| GAPDH(mice) | CATGCCTTCCGTGTTCCTA | CTTCACCACCTTCTTGATGTCATC |
| ERβ(Rat)  TPH2(Rat)  GAPDH(Rat) | TCACCGTGGAGCCTTAGTTC  TTGGGAGGTGGTTTCTACTTTC  GACATCAAGAAGGTGGTGAAGC | TCTGCATAGAGGAGCGATGA  TGTTTCTCTGTGACTCGGTTTC  TGTCATTGAGAGCAATGCCAGC |
