# Supplementary figures and images for: Ahi1 regulates serotonin production by the GR/ERβ/TPH2 pathway involving sexual differences in depressive behaviors
Source: Cell Commun Signal. 2022 May 28;20:74. doi: 10.1186/s12964-022-00894-4 (PMC9148486; doi:10.1186/s12964-022-00894-4)

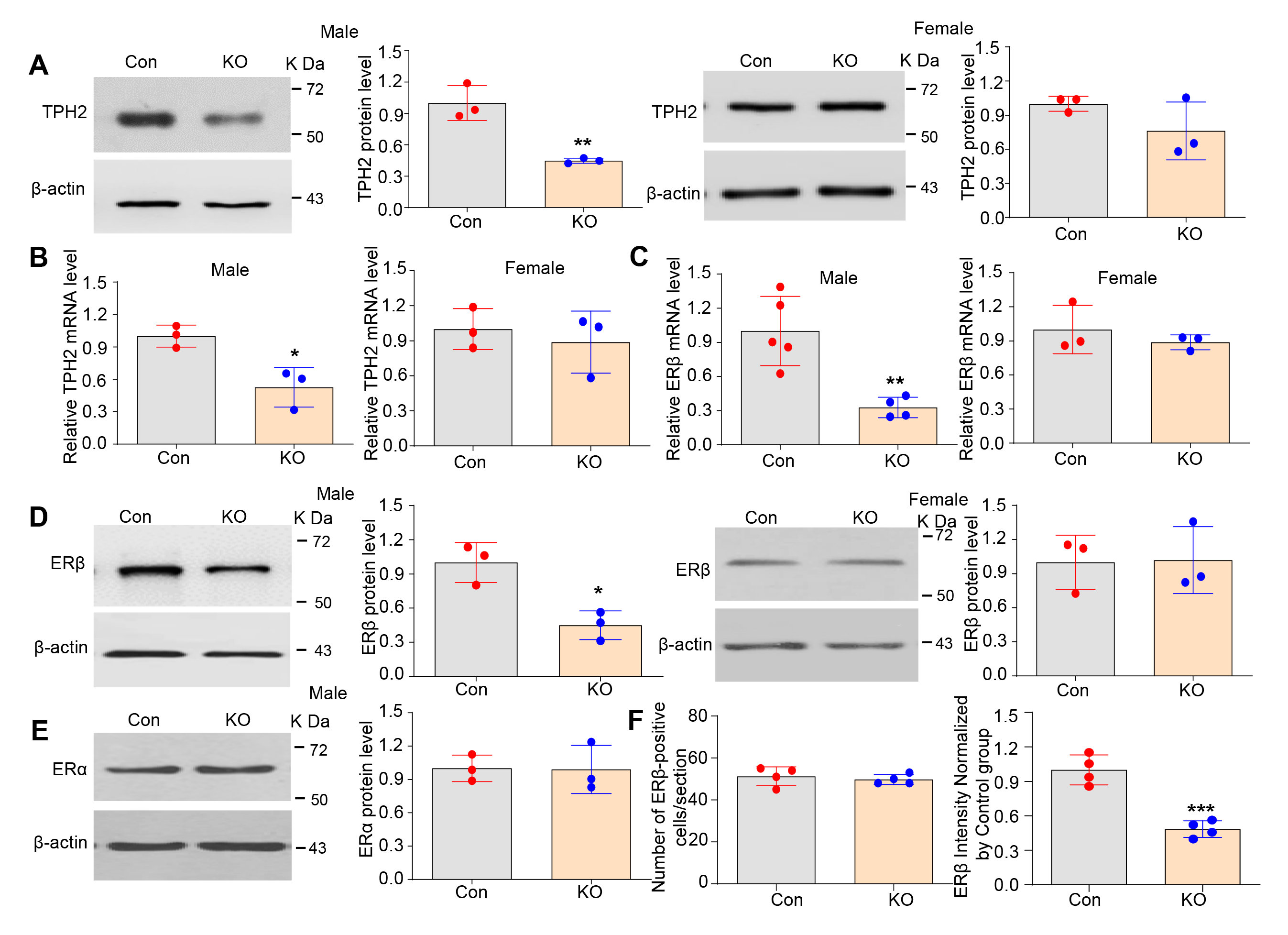

Supplement: Supplementary file 3 — Additional file 2: Fig. S1. There were sex differences in ERβ/TPH2/5-HT pathway and depression-like behaviors in the brainstem of Ahi1 KO mice. A After female and male Ahi1 KO mice were sacrificed, brainstem tissues were collected, and TPH2 protein expression was examined by Western blot analysis. N=3. B TPH2 mRNA levels were quantified by quantitative PCR in the brainstem tissues of female and male Ahi1 KO mice. N=3. C ERβ mRNA expression in brainstem tissue of female and male Ahi1 KO mice was examined by quantitative PCR. N=3-5. D ERβ protein expression in brainstem tissue of female and male Ahi1 KO mice was examined by Western blot analysis. N=3. E ERα protein expression was examined in hippocampus tissue of male Ahi1 KO mice by Western blot analysis. N=3. F Quantity analysis of ERβ expression fluorescence intensity and the number of ERβ-positive cells in the brainstem in male Ahi1 KO mice. *p<0.05, **p<0.01, ***p<0.001 versus Control. [file 12964_2022_894_MOESM3_ESM.tif]

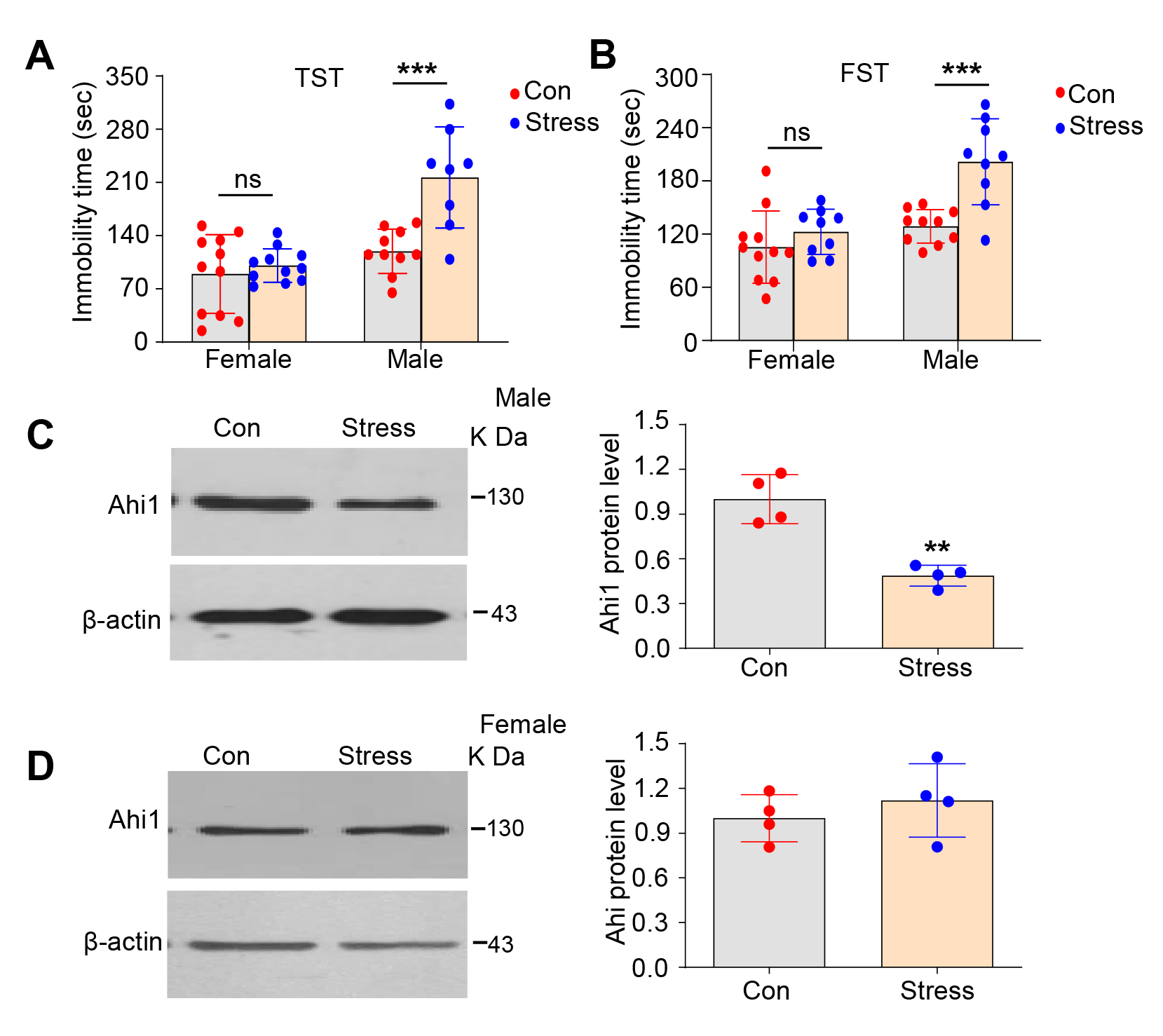

Supplement: Supplementary file 4 — Additional file 3: Fig. S2. Stress led to depression-like behaviors and a decrease of Ahi1 in the hippocampus of male mice, but not in female mice. A, B After the healthy female and male C57 mice were stressed for 2 weeks, the behavioral tests were performed in the stressed mice. the immobility time in TST and FST tests was recorded. N=8-11. C, D After female and male C57 mice were stressed for 2 weeks, the hippocampal tissues of male and female stressed mice were collected and their Ahi1 content was detected by Western blot. N=4. **p<0.01, ***p<0.001 versus Control). [file 12964_2022_894_MOESM4_ESM.tif]

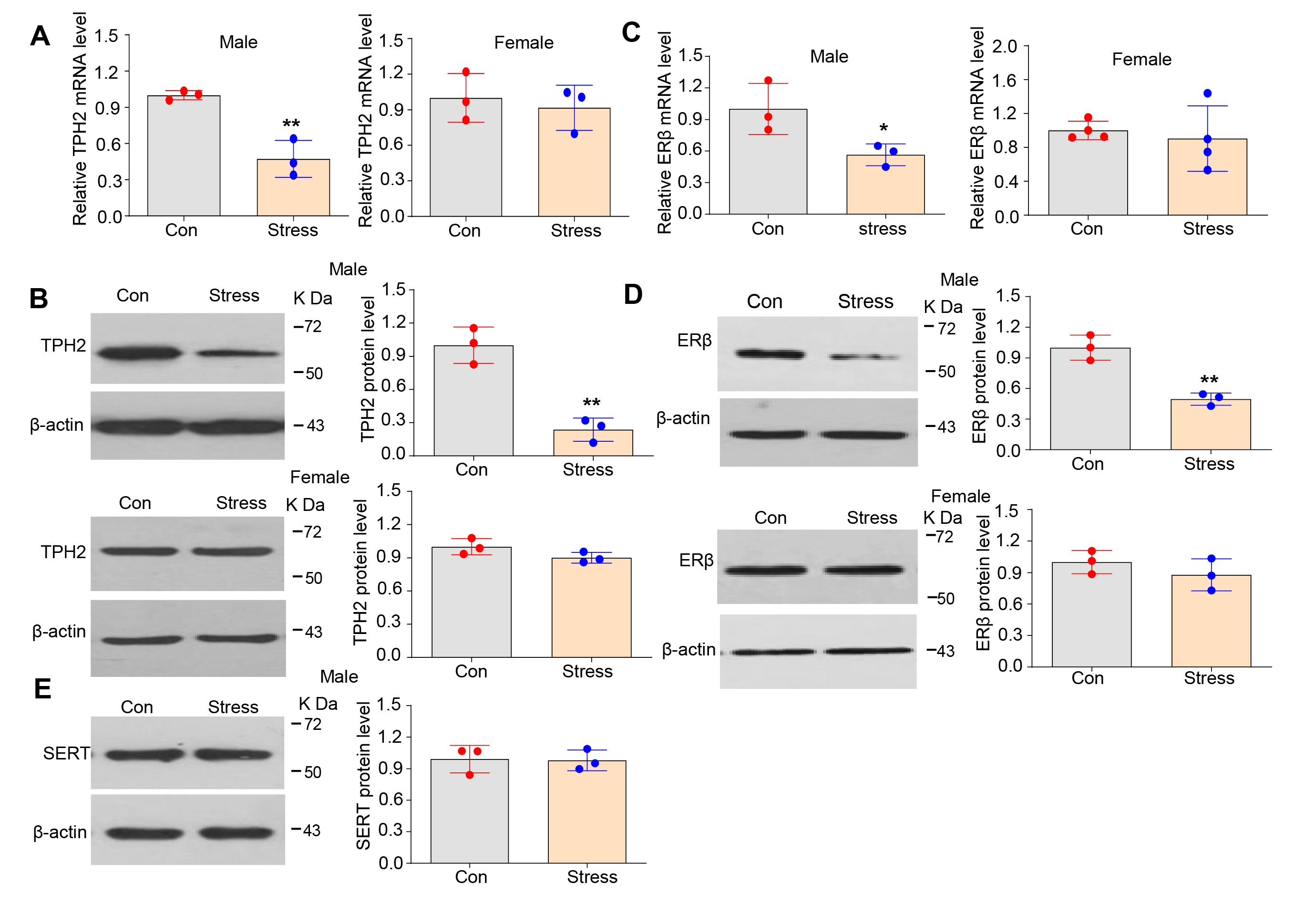

Supplement: Supplementary file 5 — Additional file 4: Fig. S3. There were sex differences in ERβ/TPH2/5-HT pathway in stressed mice. A, B After female and male stressed mice were sacrificed, hippocampus tissue was collected, TPH2 mRNA levels were quantified by quantitative PCR (A) and TPH2 protein expression was examined by Western blot analysis (B). N=3. C ERβ mRNA levels were quantified by quantitative PCR in the hippocampus tissue from female and male stressed mice. N=3-4. D ERβ protein expression was performed in hippocampus tissue from female and male stressed mice. N=3. E SERT protein expression was examined in the hippocampus tissue of male stressed mice by Western blot analysis. N=3. **p<0.01 versus Control. [file 12964_2022_894_MOESM5_ESM.tif]

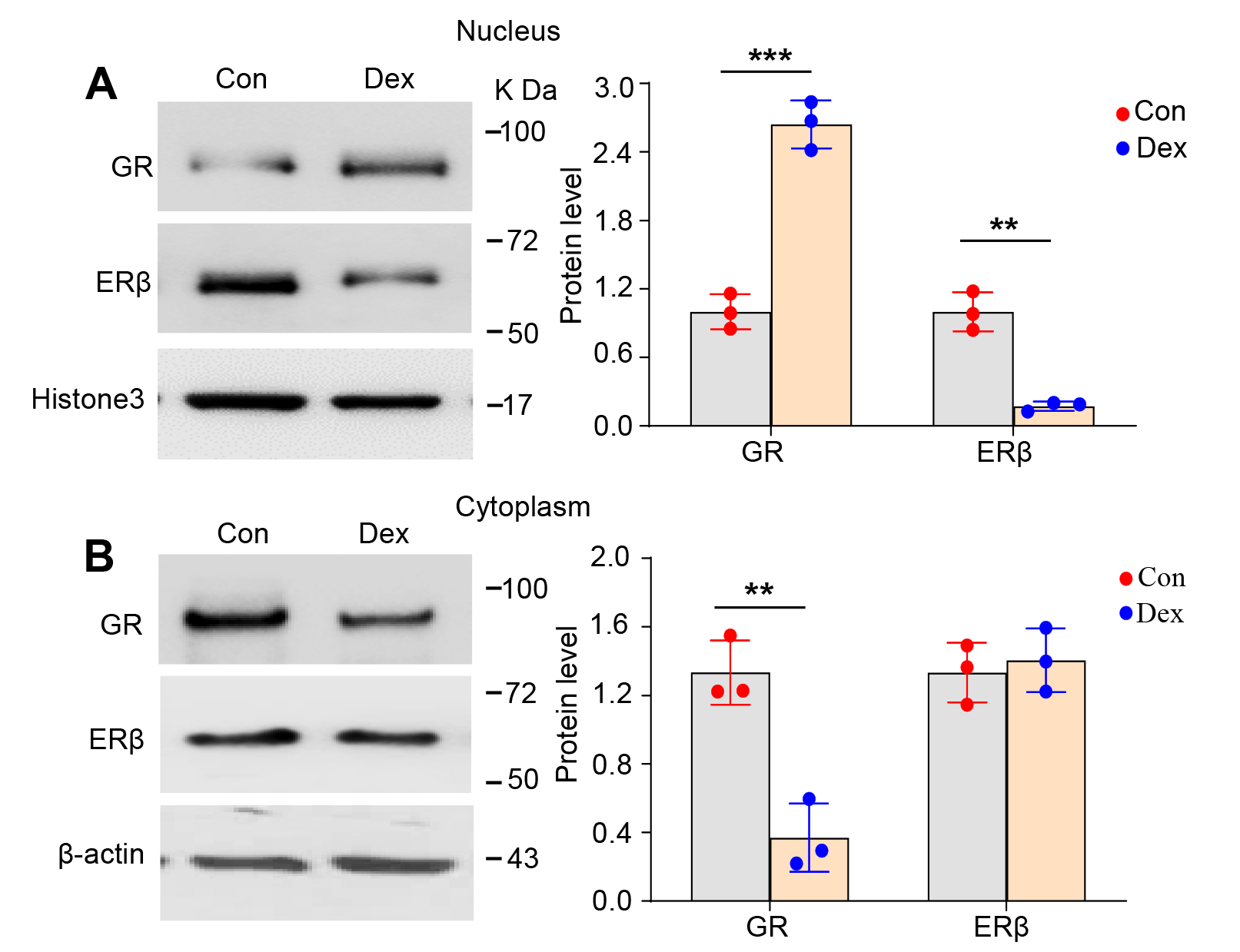

Supplement: Supplementary file 6 — Additional file 5: Fig. S4. Dexamethasone promotes GR nuclear translocation and inhibited the transcription of ERβ mRNA. ERβ and GR protein expression were detected in the nucleus (A) and cytoplasm (B) in Dex-treated PC12 cells for 72 h by Western blot analysis. N=3. **p<0.01, ***p<0.001 versus Control. [file 12964_2022_894_MOESM6_ESM.tif]
